# Patterns of Cancer Incidence and Mortality in North- Eastern India: The First Report from the Population Based Cancer Registry of Tripura

**DOI:** 10.31557/APJCP.2020.21.9.2493

**Published:** 2020-09

**Authors:** Shreya Sarkar, Dhritiman Datta, Shiromani Debbarma, Gautam Majumdar, Syam Sundar Mandal

**Affiliations:** 1 *Department of Epidemiology and Biostatistics, Chittaranjan National Cancer Institute, Kolkata, India. *; 2 *Dalhousie Medicine New Brunswick, Saint John, NB, Canada. *; 3 *New Brunswick Heart Centre, Saint John Regional Hospital, Saint John, NB, Canada. *; 4 *Regional Cancer Centre, Agartala, Tripura, India. *

**Keywords:** Cancer registration, control, prevention, epidemiology, North, Eastern India

## Abstract

**Background:**

There is, till date no population-based data regarding cancer patterns in North- Eastern India, dictating the need to understand the epidemiology of cancer in this population for its effective management.

**Methods:**

This is the first report of the Population Based Cancer Registry (PBCR) in Tripura (2010-2014). The protocol involves active collection of data on all cancer cases from Tripura through staff visit in more than 150 sources of incident and mortality registration, government and private hospitals, municipal corporation, etc. and scrutiny, corroboration with existing records. Data was analyzed statistically to understand cancer trends in terms of incidence and mortality across different sites, age groups affected and gender.

**Results:**

A total of 10,251 cases were registered during the period, with overall age-adjusted incidence rates of 75.7 and 54.9 per 100,000 males and females respectively. Crude Incidence Rate (CR) and Age- Adjusted Rate (AAR) was among the lowest reported in India, probably due to associated socio-economic factors. The most prevalent cancers were lung (18.1%), esophageal (8.3%) for men and cervix uteri (17.6%), breast (13.8%) for females. Gall bladder cancer in females was one of the highest in the country. Rate of cancer mortality in the population was quite high and significantly increased with time, probably accounting for dearth in early detection and feasible treatment alternatives.

**Conclusion:**

The data suggests that high cancer incidence and mortality are prevalent in the population of Tripura, dictating the need of active tobacco control measures, early detection and awareness drives for effective cancer control.

## Introduction

The National Cancer Registry Programme (NCRP) was introduced by Indian Council of Medical Research (ICMR) in 1981 and currently holds a network of 28 Cancer Registries across the country. However, initiatives taken in North- Eastern India is relatively recent, leading to lack of reliable population- based cancer data from this part of the country. According to the latest census in India in 2011, the Indian population has already crossed 1.2 billion and not only comprises of an immense versatility in ethnicity, culture, language and other socio- economic parameters, but also in terms of distribution and patterns of both chronic and infectious diseases.

The Cancer Atlas Project was established in 2006 by NCRP, which was subsequently recognised as a Population Based Cancer Registry (PBCR) in 2009 at the prestigious Regional Cancer Centre (RCC), Agartala, to cater to cancer data collection from the entire state of Tripura. PBCR specifically focus on the cancer disease burden in terms of incidence and survival in a population residing in a specific geographic area and use of the data for evaluation and planning of disease management (Jensen et al., 1991).

The state of Tripura is located in the north- eastern part of the country between the latitudes 22056’, 24032’ (North) and longitudes 91009’, 92020’ (East). The altitude at the capital of Tripura, Agartala, is 12.80 m. Tripura has a strategic location in that it shares about 84% of the total perimeter of international border with Bangladesh. Although relatively small in terms of size (10,491.69 sq.km), 60% of the area is hilly and forested 27% is cultivated. The population is over 3.5 million with 960 females per 1000 males and has a high overall literacy rate of 87.2 %. The population is multi- religious, although pre- dominantly Hindus (85.6 %) and multi- lingual, although the most common language is Bengali. Interestingly, Tripura houses diverse ethno-linguistic tribal groups with Bengali culture co-existing with Tribal traditional practices.

Till date, there are very few reports regarding the cancer scenario in different parts in India and to the best of our knowledge, no comprehensive reports from the north- eastern part of the country. Data on cancer incidence and mortality were collected for a 5- year period from 2010- 2014 to understand the trends in this part of the country. Our observations indicate that cancer incidence and mortality was quite high in Tripura, although the overall 5- year trend of all cancers remained similar, dictating the need for effective tobacco regulation, cancer screening and control in this part of the country and in India in general.

## Materials and Methods

*Sources of registration*

Cancer is not yet a notifiable disease in India and hence, registration is not voluntary. Thus, data was collected through active participation of social workers through visits to different sources and by interviewing patients/ attendants and scrutinizing medical records. The main registration source for cancer patients of Population Based Cancer Registry, Tripura, is the Regional Cancer Centre, Agartala (86.6- 97.1% cases from 2010- 2014), due to its being the lone State hospital of cancer treatment in Tripura where the PBCR is also located. There are however more than 150 sources for Incident, Mortality registration in the whole state including Government hospitals (121), private hospitals (1), nursing homes and polyclinics (12), private pathological laboratories and radiological centres (25), Death, Birth Registry, Municipal Corporation, Crematory, Village Panchayet, etc. Majority of the mortality data was obtained from social investigators during their home visit activities apart from their normal visits of hospitals, crematory, death, birth registry office etc. A total of 10,251 incident cancer cases were registered during the period from 2010 to 2014. Clearance from the institutional ethical committee was obtained prior to the study, as well as written informed consent from all participants.

*Parameters for collection of data*

Primary site and histology were categorised according to International Classification of Diseases for Oncology (ICD) (Jensen et al., 1991). All clinic-pathological and demographic data were manually and electronically recorded, including identifying information of the patient, residential address, duration of stay at permanent address, date of first diagnosis, diagnosis status, ICD coding, etc. Informed consent was obtained from all the patients prior to the study. The data was then matched with the incident database of RCC, Agartala and PBCR. Data on mortality was collected from previously mentioned sources to include details and matching of mortality data with morbidity cases was carried out (Fritz et al., 2013). Non- matched death cases and those with no case history, other than the availability of death certificate were registered as “Death Certificate Only” (DCO) and entered into the database along with ICD coding (flow chart in Supplementary Figure 1, Supplementary Table 1). All new data were thoroughly verified with those existing in the registry to avoid duplication.

The registry is reported in the span of five years from 2010-2014 and reported in terms of sex, site, Crude Incidence rates (CR), Age- Adjusted Incidence Rates (AAR) and Truncated Rate (TR) per 100,000 persons and directly using world standard population (Sen et al., 2002). 

*Statistical analysis*

Chi square for trend (Extended Mantel Haenszel) was used to detect the linear variation with subsequent time and p value was used to understand significance (Sarkar et al., 2017). All tests were two tailed, with a Confidence Interval (CI) of 95% and probability (p) value <0.05 being considered significant. Calculations were by softwares Epi Info 7 (CDC, Atlanta).

## Results

*Males presented with higher pre- disposition to risk and incidence the disease*

**Figure 1:**
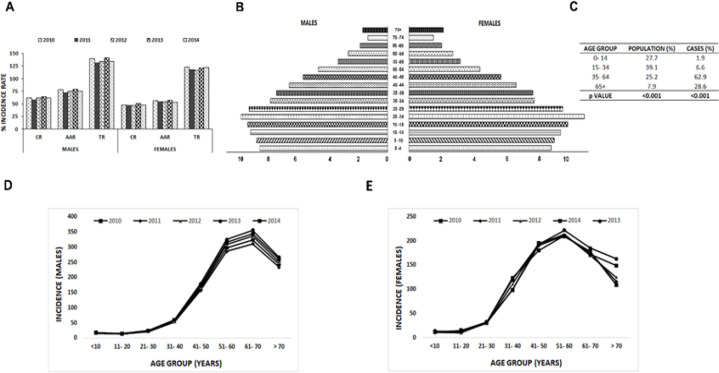
Pattern of Cancer Incidence from the 5- Year Period of 2010- 2014. A, crease with time; B, Population pyramid showing distribution in 5- year age groups according to ICMR estimate of the years 2012- 2014; C, Cancer incidents in 5- year age groups in the years 2010- 2014; D, Trend in cancer incidents 10-year age groups in males; E, Trend in cancer incidence in 10-year age groups in females. Abbreviations: CR, Crude Rate; AAR, Age- Adjusted Rate; TR, Truncated Rate

According to the 2011 census, the population of Tripura was 36,73,917, out of which there were 18, 74,376 males. A total of 10,251 cases were registered during the 5- year period from 2010- 2014, out of which there were 5,859 males and 4,392 females (Table 1). The number of patients progressively increased over time. Males presented a higher population at risk and incidence to malignancy (Table 1). Among the reported malignant cases, males presented with higher CR than females (61.4, 47.9 respectively) (Figure 1A). Similarly, in AAR and TR, males present with a higher fraction (75.7, 136.1 respectively) compared to females (54.9, 120.2 respectively) (Figure 1A).

*Patients presented with a relatively older age of onset, although 5- year disease burden remained similar*

Due to lack of estimated distribution of the population by 5 -year age group during the census report of 2011, the data was obtained from Indian Council for Medical Research (ICMR) for the period 2010- 2014 (Figure 1B). Age group 35- 64, which represents about 25% of the population carried the maximum incidence of new malignant cases, about 60 %, indicating the significant positive correlation of cancer with the biological process of ageing (p for trend from ages 0-64 years <0.001) (Figure 1C). The population having lower age group was greater but had lower disease burden. Interestingly, the incident rate of cancer at the older age group (> 35 years) was high despite the lower percentage of individuals within that age window, indicating the correlation of cancer with increasing age (Figure 1C). The trend in cancer burden in a 10- year age group distribution remained similar in both males and females (Figure 1D, E, Table 2). However, differential pattern in age- related distribution was observed between both genders, with older men and middle- aged women presenting a higher propensity to develop cancers (Figure 1D, E).

*Specific cancers presented at a higher incidence rate but with comparable annual 5- year trends*

**Figure 2:**
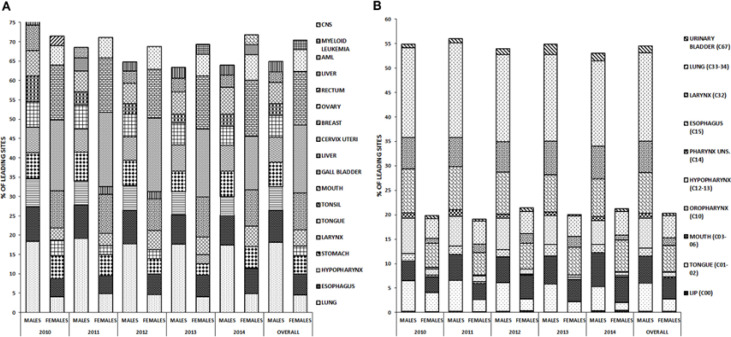
Incidence of leading cancers in different sites between males and females between 2010- 2014. A, Incidence of cancers in all males and females; B, Incidence of tobacco- associated cancers

**Figure 3:**
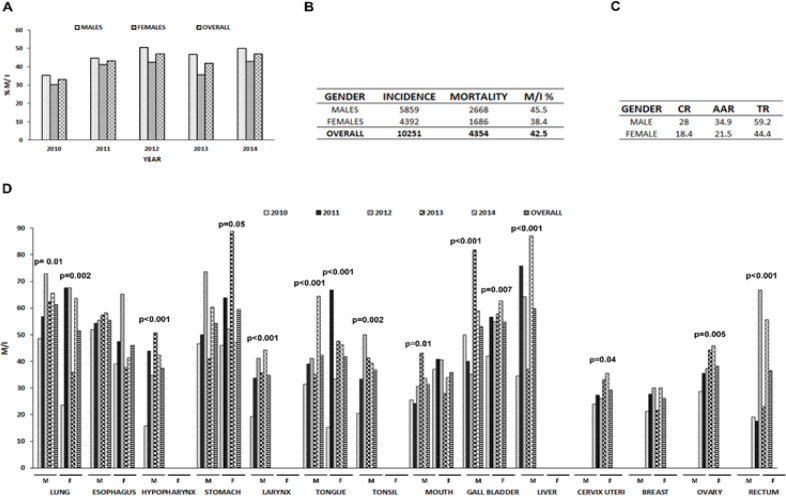
Mortality due to cancers. A, Percentage of mortality per incidence in males and females from 2010- 2014; B, Overall incidence and mortality due to cancer from 2010- 2014; C, Mortality rates in males and females; D, Mortality per incidence of leading cancers in males and females between 2010- 2014. M/I significantly increased with time for most cancers; Abbreviations: CR, Crude Rate; AAR, Age- Adjusted Rate; TR, Truncated Rate; M/I, Mortality to Incidence Ratio

The incidence of a particular cancer was similar in the time span studied (Figure 2A). Lung and esophageal cancers were the highest among men (5- year average 18.1%, 8.3% respectively), while cervical and breast cancers were the highest among women (5- year average 17.6%, 13.8% respectively) (Figure 3A), probably due to associated lifestyle and risk factors. Within the time frame studied, tobacco- related cancers were quite high among both men and women with differential distribution of the various sites affected (Figure 2B). Lung and esophageal cancer were the highest among men (18.1%, 8.3% among tobacco related cancers), while esophageal and lung were the highest among women (5.4%, 4.5 % respectively).

*High rate of mortality was observed in the population*

Cancer- related deaths among males and females was high in Tripura and in the ratio of 8:5. Deaths due to cancer was 8.2 % among all deaths. Overall mortality per incidence increased with progressing years (Figure 3A). The mortality versus incidence of cancer was higher in males, as was CR and AAR (Figure 3B, C). The incidence of mortality ratio in different cancers varied with cancer types in increasing ratios annually (Figure 4D). Among men, highest mortality was due to lung and liver cancers (5- year average M/I 61.3, 59.7 respectively), while those among women were due to stomach and gall bladder cancers (5- year average M/I 59.6, 54.8 respectively). Most cancers presented a significant rise in mortality in the 5- year period studied (Figure 3D).

**Table 1:** Population at Risk (Males and Females) in Tripura and the Malignant Cases Diagnosed between the Years 2010- 2014

Year	Popultion at Risk				Number of Malignant Cases			
	Males	Females	Total	Ratio (M:F)	Males	Females	Total	Ratio (M:F)
2010	1,857,926	1,782,253	3,640,179	10:09	1,139	846	1,985	13:10
2011	1,882,656	1,808,246	3,690,902	10:09	1,092	844	1,936	13:10
2012	1,907,714	1,834,618	3,742,332	10:09	1,177	866	2,043	14:10
2013	1,933,106	1,861,372	3,794,478	10:09	1,243	942	2,185	13:10
2014	1,958,835	1,888,522	3,847,357	10:09	1,208	894	2,102	14:10
*p* value					0.86	0.84		

M, Male; F, Female

**Table 2:** Incidence of Cancers between 2010- 2014 in Males and Females According to 10-Year Age Groups and Sex

	YEAR	2010	2011	2012	2013	2014
Age (years)		Incidence				
<10	Male	17	16	17	18	18
	Female	12	11	13	14	12
10-20	Male	14	13	14	15	14
	Female	12	10	11	13	15
21- 30	Male	23	22	23	24	24
	Female	32	31	30	33	32
31- 40	Male	55	53	58	60	59
	Female	99	99	111	120	123
41- 50	Male	164	157	169	179	173
	Female	195	190	193	192	180
51- 60	Male	298	286	309	325	316
	Female	209	211	213	222	211
61- 70	Male	324	311	336	356	344
	Female	178	176	170	185	172
> 70	Male	244	234	251	266	260
	Female	109	116	125	163	149
Total	Male	1,139	1,092	1,177	1,243	1,208
	Female	846	844	866	942	894

## Discussion

Knowledge on the trends in different cancers are essential for designing strategies for effective cancer control globally. Although cancer prognosis in terms of death and years of life lost provide valuable information, data regarding cancer incidents in the population provide valuable information to understand the risks and risk factors associated various cancers, which in turn aids in early detection and prevention.

In- depth analysis of the 5- year data of the Tripura Population- Based Cancer Registry provides an invaluable insight into the cancer patterns in this north- eastern state of India, with its unique ethnic and socio- economic setting. The data has been reported two years after promotion of the National Cancer Registry Program (established, 2006) into the Population- based one, hence eliminating most factors of under- reporting. However, those cases which were at all missed were probably in advanced cancers with dismal prognosis, especially in older patients diagnosed in outpatient clinics. These patients might not have received any curative treatment, thus eliminating medical records and hence registration. However, the authors believe that most of these cases would be efficiently recorded through DCO.

The CR and AAR, as well as TR in males and females increased with time, although the rates were quite low as compared to those obtained in other Indian registries from the period of 2012- 2014 (Males, CR range 39.9 (Barshi expanded, 2012) – 160.7 (Thiruvananthapuram District, 2012- 2014). The reason might be due to a combination of several factors: 1) low- risk lifestyle of the people in Tripura, including environmental and socio- economic parameters, 2) incomplete and inaccurate reporting of the number of patients diagnosed and treated outside the state, 3) death of patients before diagnosis and, 4) patients unwilling of confirmation of the malignancy due to socio- economic reasons. However, males show an increased incidence rate compared to women, probably accounting for associated etiological factors such as usage of tobacco, alcohol, occupational exposure, etc. (Sinha et al., 2003; Das et al., 2015). 

The overall 5- year age group disease burden remained similar in Tripura, the trend being similar to that observed in other cities according to the PBRC report, 2012- 2014. Interestingly, although about 25% of the population belong to the age group of 35- 64 years, the maximum incidents of cancer, i.e., 63% occur in this group, indicating a significant association between ageing and cancer. Similar results have been previously shown in other studies (SEARO, 2004; Sharma and Radhakrishnan, 2011; Mallath et al., 2014; Thakkar et al., 2014). 

The incidence rate varied between different cancers and between males and females, the results being similar with that obtained in other Population Based Cancer Registries. Lung and esophageal cancers were the highest among men, primarily due to habits such as smoking and chewing tobacco, alcohol consumption, etc (Domper Arnal et al., 2015; Malhotra et al., 2016). Similar high incidence of lung cancer has also been reported in other parts of the country and globally (Islami et al., 2015; Malik and Raina, 2015; Noronha et al., 2016). In women, cervix uteri and breast cancers presented the maximum disease burden, being associated factors such as infection with Human Papillomavirus (HPV), poor hygiene and awareness, diet and exercise, genetics, etc. (Hankinson et al., 2004; Haverkos, 2005; Crosbie et al., 2013) In recent studies, high risk HPV has also been associated with the pathogenesis of breast carcinoma (Lawson et al., 2015). High incidence of cervical cancer has similarly been reported in many studies across India (Kaarthigeyan, 2010; Sreedevi et al., 2015). Surprisingly, gall bladder cancer in females (9%) is present at a percentage higher than reported in most parts of the country (PBCR Report, 2012- 2014), only comparable to Cachar District (10.3%), Dibrugarh District (10.7%), Kamrup Urban District (9.3%).

Tobacco usage, both in smoking and smokeless forms is linked to a variety of cancers (Kuper et al., 2002; Vineis et al., 2004). Widespread usage of different forms of tobacco, both in men and women (mostly in smokeless form) (Mishra et al., 2015; Nair et al., 2015) accounts for the high prevalence of tobacco associated cancers observed in our study. Several reports, both within Tripura and other parts of India and across different age groups indicate the widespread use of tobacco in our country (Rani et al., 2003; Sinha et al., 2003; SEARO, 2004; Pal and Tsering, 2009; Agrawal et al., 2013; Das and Baidya, 2014; Mishra et al., 2016). Although several Indian legislations for control of tobacco usage exist in India, such as the National Tobacco Control Programme and Tobacco Cessation Services, further stringent regulation needs to be enforced to minimize tobacco associated health hazards (Shimkhada and Peabody, 2003; Mishra et al., 2012).

Mortality rate reported in our study was quite high among cancer patients (Overall 5- year M/I= 45.2% for males, 38.2 for females) and showed and increasing trend with time. This rate was much higher than those reported my most PBCRs across the country (M/I % for Males: Range 10.1(Delhi)- 68.9 (Barshi Rural), Females: Range 8.0 (Delhi)- 66.3 (Barshi Rural) and showed a significant increment with time in most types of cancers. The ratio of male to female deaths was 8:5. Lung cancer, which was the most prevalent form of cancer in men was also the leading cause of cancer- related mortality, while stomach cancer ranked the highest among mortality in women. A combination of several factors could account for this high mortality rate- lack of optimal treatment facilities in rural regions, financial constraints of less affluent individuals, lack of screening programs, late stage diagnosis, etc. (Mallath et al., 2014; Tripathi et al., 2014; Gupta et al., 2015). Similar mortality reports on developing countries were reported both in India and globally with data indicating a rise in cancer associated mortality rates in the future (Jemal et al., 2011; Dikshit et al., 2012).

In conclusion, the Tripura Population Based Cancer Registry, despite having certain limitations in terms of quality of data, due to its being in its early stages, provides comprehensive information on the patterns of cancer in the north- eastern part of India. These observations, which provide valuable information over a 5- year period could be used not only to understand the epidemiology and etiology associated with various cancers, but also aid in effective cancer control in this region. Legislatures and awareness programs to control tobacco usage could reduce the disease burden of several tobacco- associated cancers. Early detection, along with a healthy lifestyle, vaccination programs, physical examination and health education should be encouraged in women for control of cervical and breast cancers. These results could act as a useful guide for designing and implementation of cancer control programs and treatment facilities in this region.
